# Assessment of the Physical and Energetic Properties of Fuel Pellets Made from Sage Waste Biomass with the Addition of Rye Bran

**DOI:** 10.3390/ma16010058

**Published:** 2022-12-21

**Authors:** Krzysztof Jadwisieńczak, Sławomir Obidziński, Dariusz Choszcz

**Affiliations:** 1 Department of Heavy Duty Machines and Research Methodology, University of Warmia and Mazury in Olsztyn, 10-957 Olsztyn, Poland; 2 Department of Agri-Food Engineering and Environmental Management, Bialystok University of Technology, 15-351 Białystok, Poland

**Keywords:** sage waste biomass, pellets, rye bran, energetic properties

## Abstract

The aim of the present study was to evaluate the effect of rye bran addition on the pelleting process of sage waste biomass, and the quality and energetic properties of fuel pellets. The pelleting process was conducted on an SS-4 test stand equipped with a P-300 pelletizer with flat die roller compactors. The addition of 20% rye bran reduced the pelletizer’s power/energy consumption from 3.75 kW/107 kWh t^−1^ (0% rye bran content) to 3.19 kW/91 kWh t^−1^, decreased physical and bulk density, and increased the pellet durability index (PDI). The higher heating value—HHV (19.39 MJ kg^−1^ at 10% humidity) and the lower heating value—LHV (18.17 MJ kg^−1^) of sage waste biomass indicate that this plant material is highly suitable for heat generation. The addition of 20% rye bran decreased HHV by 2.07% and LHV by 2.67%.

## 1. Introduction

The depletion of fossil fuels necessitates the search for more innovative sources of energy. Renewable energy sources, including wind, solar radiation, and geothermal energy, are gaining increasing popularity. However, the use of biomass as a source of renewable energy has recently attracted the greatest attention. Various forms of biomass (wood waste, energy crops, grasses, straw) have been used as an energy source for many years. The suitability of biomass for energy generation has been confirmed by numerous research studies.

According to the International Renewable Energy Agency [1], bioenergy accounts for around three quarters of the world’s renewable energy consumption, of which more than half consists of traditional biomass use. The “Global Status Report” [2] estimates that biomass will continue to be the main source of renewable energy, with a share of nearly 60% by the year 2030. According to https://www.eia.gov [3], accessed on 2 June 2021, biomass provided about 4835 trillion British thermal units (TBtu), or about 4.8 quadrillion Btu and equal to about 5% of total U.S. primary energy consumption. Of that amount, about 2316 TBtu were from biofuels (mainly ethanol), 2087 TBtu were from wood and wood-derived biomass, and 431 TBtu were from the biomass in municipal solid wastes and sewage, animal manure, and agricultural byproducts. The amounts—in TBtu—and percentage shares of total U.S. biomass energy use by consuming sector in 2021 were: industrial—2313 TBtu—48%, transportation—1477 TBtu—31%, residential—464 TBtu—10%.

Cereal straw, mainly wheat straw, is widely used as feedstock for bioenergy generation. The suitability of compacted cereal straw for energy purposes was investigated by Azócar et al. [4], Obidziński [5], Serrano et al. [6], and other researchers. Wood waste, including sawdust, bark, and logging waste, can also be used as feedstock for energy production [7,8,9,10]. Other examples of biomass feedstocks include garden waste [11], food processing waste [12,13], and herbal waste.

According to Kobus et al. [14] and Kumar et al. [15], herbal waste is generated during harvesting, drying, transport and storage of herbal biomass. Plants that are broken down into very small fractions during these processes are not suitable for industrial processing and are regarded as waste. However, these fractions can still contain significant amounts of essential oils that are a rich source of many biologically active substances and fragrances [16,17].

Herbal waste can be converted to liquid fuel [14] and biogas [16,18,19]; it can be used in catalytic pyrolysis [20] and in the production of compost [21] and vermicompost [22,23]. Herbal and food wastes [24] can be used as feed additives [15,17,25], in the production of packaging [26], as organic substrates in cucumber production [27], or in the production of solid fuels [28,29].

Most herbal waste biomass is processed into feed additives. Kisworo et al. [30] analyzed the potency, physicochemical characteristics, and the content of secondary metabolites in solid herbal waste as a source of fiber and nutrients in ruminant diets. Zhuang et al. [31] found that diets where the forage component was replaced in 30% by fermented herbal tea residues induced changes in gut microbiota, delivered health benefits, and alleviated heat stress in beef cattle. Kowczyk-Sadowy et al. [32] evaluated the feed value of a mixture of pelletized couch grass rhizomes with 15%, 20%, and 25% addition of potato pulp. According to Teixeira et al. [33], *Moringa oleifera* leaf meal, which is widely available in many tropical countries, is also a good source of antioxidant compounds such as ascorbic acid, flavonoids, phenolics, and carotenoids. Kongmun et al. [34] reported that garlic is an effective alternative growth promoter in livestock production that improves the growth rate of animals, nutrient digestibility, and carcass traits. Lemongrass and peppermint are used as feed additives to improve the performance of beef and dairy cattle [35]. Menthol (*Mentha arvensis*) enhanced ileal protein and amino acid digestibility and improved feed efficiency in weaned piglets [36], whereas black paper improved the performance of broiler chickens [37].

Herbal waste can also be managed by composting [38,39,40]. According to Zhou et al. [41,42] and Greff et al. [43], co-composting with herbal wastes increases the rate at which organic matter (OM) is degraded by microorganisms and leads to the production of high-quality compost with antipathogenic properties. Greff et al. [43] investigated the influence of *Cellulomonas favigena* and *Streptomyces viridosporus* bacterial inoculants on the compostability of post-extraction lavender waste and found that lavender waste enhanced the composting process by extending the thermophilic phase, accelerating OM degradation, and increasing the viable counts of beneficial microorganisms. However, adverse effects were also observed.

In Poland, herbal plants are grown on an area of 25,000 ha [44]. Around 60 species of herbal plants are currently cultivated in these areas [45]. Sixteen species are in high demand in the processing sector and on the consumer market, and their production continues to increase. These include plantain, peppermint, savory, angelica, St. John’s wort, fennel, valerian, marjoram, sage, and milk thistle [46]. Depending on the properties of a given part of the plant (leaves, flowers, roots, fruit), they have different uses in pharmaceutical, food and, cosmetic industries. Some plant parts are not recyclable and are treated as waste which is either recycled or composted. Herbal production waste can be also used as feedstocks for agricultural biogas plants to produce energy and digestate which is a valuable agricultural fertilizer [47]. However, plant-based waste can be a more problematic substrate for biogas plants than animal manure. Plants containing cellulose and lignin are particularly difficult to degrade, and these compounds may decrease the effectiveness of anaerobic digestion in biogas plants [48].

Sage is a widely cultivated herbal plant, and waste biomass is produced mainly during harvest. Sage has antimicrobial and antioxidant properties, and it used in the production of pharmaceuticals and cosmetics [24,25,26]. Sage waste biomass, including straw and stems, has a high heating value [49] and can be processed into pellets [19,50].

In recent years, waste biomass has become a popular feedstock for heat generation. Gill et al. [51] studied the effect of moisture content and particle size distribution on the quality of rice straw pellets. Atabani et al. [52] produced and burned pellets made from spent coffee grounds and other waste biomass. Blancarte-Contreras [53] investigated the mechanical and energetic properties of pellets produced from agave fiber mixed with Pinus species sawdust in six different proportions. Chojnacki et al. [54] added wastes from the production of apple, carrot, and red beet juice to barley straw and examined their influence on the density, hardness, ash content, and calorific value of pellets. Kulokas et al. [55] investigated the properties of pellets produced from straw with the addition of buckwheat hulls. Carrillo-Parra et al. [56] produced pellets from a mixture of oil palm residues (at a ratio of 100:0, 80:20, 60:40, 40:60, 20:80, and 0:100) and pine sawdust, tested their physical and mechanical properties, and the impact of pine sawdust on the chemical composition of oil palm residue mixtures. Dołżyńska et al. [13] examined the properties of pellets containing plum biomass. Stolarski et al. [57] investigated the energetic properties of pellets made from *Coppice willow* and *Virginia mallow* biomass, and Stasiak et al. [8] analyzed the mechanical and combustion properties of pellets with different proportions of straw. Gonzalez et al. [58] pelletized garden waste (fallen leaves) with different moisture (10, 15 and 20 wt%) and glycerol (binder) content (0, 5, and 10 wt%) and examined the physical and thermochemical properties of the obtained pellets. Szyszlak-Bargłowicz et al. [59] analyzed the energy parameters of miscanthus biomass, copra cake, their blends, and the resulting pellets, as well as energy consumption during the pressure agglomeration process.

Bran is composed of the pericarp, the aleurone layer, and the seed coat, with an admixture of ground germ and endosperm [60]. Bran can be generally divided into two groups: light bran with a low content of the endosperm and a high content of the pericarp and the seed coat, and whole bran with a high content of the endosperm and a low content of the seed coat [60]. Bran accounts for 10% of rye grain, and it is separated during the milling process [61].

According to Kamal-Eldin et al. [62], rye bran contains 41–48% dietary fiber and 13–28% starch on a dry matter basis. The ash content of rye bran does not exceed 2.8%. Rye bran is also more abundant in protein (14–18%) than rye flour (5–16%) [63].

Due to its high content of dietary fiber, rye bran is used in the production of dietary supplements to stimulate peristalsis and decrease the energy density of the diet (by increasing and prolonging satiety) [64].

Rye bran is also an effective binder, and it is applied in granulation and compaction processes to decrease energy consumption and improve the quality of pelleted materials [65,66,67].

The aim of the present study was to determine the effect of rye bran addition (10% to 20%) on the pelletization of sage waste biomass and the quality of the obtained pellets (including their energetic properties). Ideally, the biofuel production process should be energy-efficient; therefore, an attempt was made to analyze the suitability of an easily available additive, i.e., food processing waste, for energy generation. The results of the study can be used to optimize the proportions of rye bran in pellets made from sage waste biomass, maintain desirable physical (mechanical) and energetic properties of pellets, and reduce energy consumption during pellet production.

## 2. Materials and Methods

### 2.1. Waste Biomass

**Figure 1:**
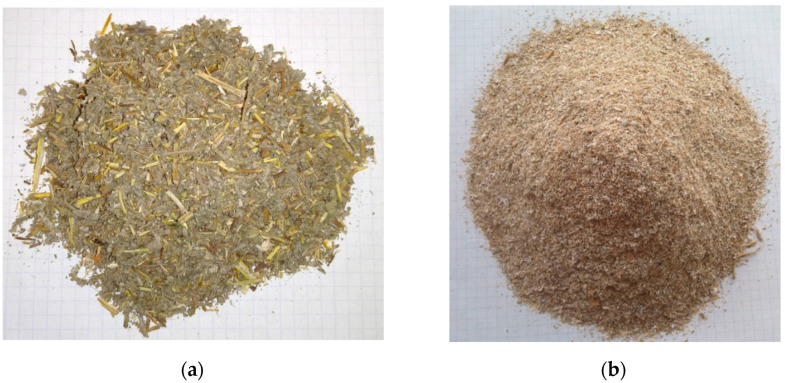
Raw materials for the study: (**a**) sage waste biomass, (**b**) rye bran.

Sage waste biomass comprising shredded stem and leaf residues (Figure 1a), and rye bran (Figure 1b) were used in this study. Sage straw waste was obtained from a private farm in Kruszyn, Warmian-Masurian Voivodeship; and rye bran was acquired from a mill in Romaszówka near Korycin in Podlaskie Voivodeship. Both voivodeships are located in north-eastern Poland.

### 2.2. Determination of Moisture Content

The moisture content of the tested raw materials and mixtures was determined using the RADWAG MA50/1. R moisture analyzer, according to standard [68]. Moisture content analyses were performed in the laboratory of the Department of Agri-Food Engineering and Environmental Management of the Faculty of Civil Engineering and Environmental Sciences at the Białystok University of Technology. Moisture content was determined in five replicates, each time by drying a sample of around 5 g at a temperature of 105 °C. The moisture content of the analyzed raw materials was determined as the average moisture content.

### 2.3. Determination of Particle Size Distribution

Biomass was shredded with a Bąk-H-8111 flail shredder equipped with a set of sieves with ϕ 4mm mesh size. The particle size distribution of the tested raw materials was determined using a MULTISERW-MOREK laboratory sieve, according to standard [69] and a previously described methodology [12]. Sieves with a mesh diameter of 8, 6, 4, 2, 1, 0.5, 0.25, 0.063 mm were used in the test. Shredded sage straw (100 g) was placed on the upper sieve (with the largest mesh diameter) and the machine was turned on for 5 min. The material remaining on the sieve was weighed on an OHAUS AX224M analytical balance.

### 2.4. Determination of Higher Heating Value and Lower Heating Value

The higher heating value (HHV) and the lower heating value (LHV) of the tested raw materials was determined according to standard [70] and a previously described methodology [12,13] in a KL 10Mn bomb calorimeter (Precyzja-Bit). The measurement is based on the complete combustion of the sample in a bomb calorimeter in the presence of oxygen and under increased pressure. The heat of combustion is determined by measuring the temperature increase in the calorimeter [71].

A pellet that was previously produced with a hand press was placed in the bomb calorimeter, and the ends of the ignition wire were wrapped around the electrodes. The calorimeter was closed, inflated, and pressurized with oxygen. The device was filled with water with a temperature that was 1 K lower than the temperature of water in the calorimeter jacket. The wires were properly attached and connected to the computer controlling the calorimeter, and the test was conducted.

The HHV *Q_s_^a^* of the tested pellet was determined in the laboratory and used to calculate the LHV by entering the content of hydrogen, moisture, ash, and sulfur into the calorimeter software. Hydrogen, ash, and sulfur content was determined in an elemental composition analysis of the tested raw materials, which is not presented in this paper. Hydrogen content was determined at 6.0% in rye bran and 4.5% in sage straw; ash content was determined at 3.78% in rye bran and 6.1% in sage straw; and sulfur content was determined at 0.33% in rye bran and 0.1% in sage straw. The calculations were performed using the formula provided in the instruction manual of the Precyzja-Bit KL 10Mn calorimeter (1):

(1) $$Q_{i}^{a}=Q_{s}^{a}-24.43\;\left(w+8.94\;H^{a}\right)\left({\mathrm{kJ}\;\mathrm{kg}}^{-1}\right)$$

where:

$Q_{i}^{a}$—lower heating value (LHV) of samples with the specified moisture content,

*W*—moisture content of the sample (%),

*H^a^*—hydrogen content of the sample (%),

24.43—coefficient that accounts for the heat of water vaporization at 25 °C in pellets with 1% water content,

8.94—coefficient that accounts for the stoichiometry of the hydrogen combustion reaction (quantitative changes).

### 2.5. Carbon and Nitrogen Content

The total content of organic carbon was determined by high-temperature decomposition with infrared detection in the Schimadzu TOC-L analyzer. A weighed sample of ground waste biomass was placed in a ceramic vessel and burned in the device. Total nitrogen content was determined by the Kjeldahl method in a Vapodest 50S device. A ground waste biomass sample of around 0.5 g was mineralized in concentrated sulfuric acid, and the resulting solution was distilled and titrated in the Vapodest 50S device.

### 2.6. Pellet Production

Pellets were produced in an SS-4 test stand equipped with a P-300 pelletizer with flat die roller compactors, according to a previously described methodology [12,13]. The stand is equipped with a vibrating conveyor that feeds the mixture into the pelletizer, and a Watt meter which measures the pelletizer’s energy consumption.

The bulk density of the sage straw and rye bran mixture in the pellet mill was determined with a 0.4 mm gap between the roller compactor and the die, at a mass flow rate of approx. 35 kg h^−1^ and a rotational speed of 280 rpm. Die openings had a diameter of 6 mm and a length of 28 mm.

The influence of rye bran addition (10%, 15%, and 20%) to sage straw on the energy consumption of the pellet mill, physical density and bulk density, and the pellet durability index (PDI) was determined in the SS-4 test stand.

### 2.7. Physical and Bulk Density

Physical density was determined by measuring the length of 10 cylindrical-shaped pellets with a caliper to the nearest 0.5 mm and by weighing the pellets on the OHAUS AX224M analytical balance. Physical density was calculated as the mass/volume ratio of 10 randomly selected pellets with the use of Equation (2). To determine the volume of pellets with a diameter of 6 mm, pellet length was measured with a caliper to the nearest 0.05 mm. Mass was determined by weighing pellets on the OHAUS AX224M analytical scale. The diameter of die openings was equal to pellet diameter (6 mm). Physical density was calculated according to formula (2):

(2) $$\rho_{g}=\frac{m}{V_{g}}$$

where:

*ρ_g_*—physical density of pellets (kg m^−3^),

*m*—pellet mass (kg),

*V_g_*—pellet volume (m^3^).

The bulk density of pellets was determined according to standard [72] by filling a container with a known volume with pellets (the top surface was leveled). The container with the pellets was weighed using an OHAUS AX224M analytical balance which was tared before the measurement. Bulk density was defined as the ratio of pellet weight to container volume.

### 2.8. Strength Properties of Pellets

The PDI was calculated according to standard [73]. During the test, pellets were passed through a 5 mm sieve to remove fine fractions, and 100 g of the pellets remaining on the sieve were placed in the Holmen NHP 100 tester and tested for 60 s. After the test, pellets were passed through the same sieve. The pellets remaining on the sieve were weighed, and the PDI was calculated. Pellets were weighted on the OHAUS AX224M analytical balance.

## 3. Results and Discussion

### 3.1. Feedstock Characteristics

**Figure 2:**
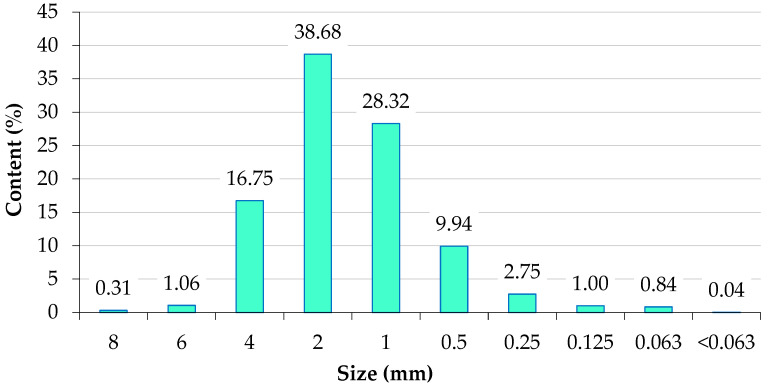
Particle size distribution of sage waste biomass.

The particle size distribution of sage waste biomass is presented in Figure 2. Particles with a diameter of 0.5, 1, 2, and 4 mm accounted for 93.7% of sage waste biomass. The proportion of fine dust particles (with a diameter below 0.5 mm) was 4.6%, and the proportion of particles larger than 6 mm was determined at 1.4%. There are no standard requirements for the particle size distribution of compacted waste biomass. It is assumed that the size of compacted particles should not exceed ½ of the diameter of die openings. The particle size distribution of sage waste biomass was suitable for pellet production in a flat die pelletizer with 6 or 8 mm mesh size.

Numerous research studies have shown that the particle size distribution of compacted materials is one of the most important parameters which influence the physical properties of pellets and the course of the pelleting process. Harun and Afzal [74] reported that the density and durability of pellets made from different types of woody biomass and their blends increased with a decrease in the particle size of ground materials. Jacinto et al. [75] found that the ideal particle size distribution for pelleting lignocellulosic biomass in a pelletizer with a flat die press (identical to that used in the present study) was 80% of particles with a diameter of ≤3.15 mm, and minimum 5% of particles with a diameter larger than 3.15 mm. Pradhan et al. [76] investigated the effect of biomass (garden waste) milling size on the pelletization process and pellet quality, and found that specific energy consumption decreased from 141.2 to 100.2 kWh ton^−1^ when biomass milling size was reduced from coarse to fine. Stelte et al. [77] observed that a decrease in biomass particle size (from 2.8 mm to less than 0.5 mm) increased pelletizing pressure due to higher friction. In a study by Mani et al. [78], the density of wheat straw, barley straw, corn stover, and switchgrass pellets increased in most cases when milling size was reduced from 3.2 to 0.8 mm. Similar results were reported by Carone et al. [79] who found that the density and durability of pellets made from pruning residues increased at smaller mill screen sizes 4, 2, and 1 mm).

The HHV and the LHV of sage straw and rye bran are presented in Table 1 and Table 2, respectively. The moisture content analysis confirmed that sage straw was adequately prepared for pressure compaction. The average moisture content from five measurements was 10.1%. The average HHV of sage straw (with 10.1% moisture content) was 19.396 MJ kg^−1^, and the LHV of sage straw (with 10.1% moisture content) was 18.169 MJ kg^−1^. An increase in moisture content decreases both LHV and HHV, as demonstrated by the results in Table 1, where both parameters were higher in samples with 0% (dry matter) than 10% moisture content.

The energy parameters of rye bran were determined in samples with an average moisture content of 13.2%. The results are presented in Table 2. The average HHV of rye bran was 16.778 MJ kg^−1^, and the LHV of rye bran was 15.124 MJ kg^−1^.

Similar values of HHV and LHV were reported in the literature for other types of biomass. In the work of Jasinskas et al. [80], the lower calorific value of three cannabis varieties with a moisture content of 9.98% to 8.87%, were 17.37 MJ kg^−1^ and 16.93 MJ kg^−1^ DM, respectively. Miranda et al. [81] determined the lower calorific value of pelleted maize cobs at 15.68 MJ kg^−1^. In a study by Gageanu et al. [82], the lower calorific value of pellets produced from a mixture of wheat straw and corn cobs with a moisture content of 8.37% was determined at 16.24 MJ kg^−1^. According to Nakomcic-Smaragdakis et al. [83], apple pruning residues (14–15% moisture content) had a net calorific value of 14.15 MJ kg^−1^.

Based on the results presented in Table 1 and Table 2, formulas for converting the HHV and the LHV of sage waste biomass and rye bran depending on their moisture content were developed (3–6):

- HHV of sage waste biomass:

(3) $$Q_{ss}=-0.2159\;w_{s}+21.555$$

- LHV of sage waste biomass:

(4) $$Q_{is}=-0.2293\;w_{s}+20.465$$

- HHV of rye bran:

(5) $$Q_{sb}=-0.1960\;w_{b}+19.357$$

- LHV of sage waste biomass:

(6) $$Q_{ib}=-0.2023\;w_{b}+17.786$$

where:

*Q_ss_*, *Q_sb_*—HHV of sage straw and rye bran (MJ kg^−1^),

*Q_is_*, *Q_ib_*—LHV of sage straw and rye bran (MJ kg^−1^),

*w_s_*, *w_b_*—moisture content of sage straw and rye bran (%).

The moisture content of the analyzed material is entered into the program to calculate the higher heating value (HHV) and the lower heating value (LHV). The program calculates HHV and LHV values for the determined moisture content and for dry weight with 0% moisture content. Therefore, the equations for calculating HHV and LHV are derived for the analyzed moisture content and for 0% moisture content.

Various formulas for calculating HHV/LHV have been proposed in the literature, depending on the parameters of the analyzed materials. The formulas applied in this study are based only on the moisture content of the examined materials.

The results presented in Table 3 were calculated based on HHV and LHV values that had been previously determined in pure samples of rye bran and sage waste biomass.

Formulas (3) to (6) were also used to calculate the HHV and LHV of sage straw pellets with different proportions of rye bran (15% moisture content). The results are presented in Table 3.

The results presented in Table 3 were calculated based on HHV and LHV values that had been previously determined in pure samples of rye bran and sage waste biomass. The obtained values show that rye bran had a minor influence on the HHV and LHV of the mixtures. These results indicate that the addition of rye bran only slightly reduced the energy values of the produced pellets. For example, 10% addition of rye bran to sage straw reduced the HHV and LHV by around 0.190 MJ kg^−1^ (approx. 1%).

Binder addition exerted similar effects on the HHV and LHV of the obtained pellets in other studies [5,84,85]. Gendek analyzed a mix of pine sawdust and pine cones, and found that the LHV ranged from 17.98 MJ kg^−1^ for pure pine sawdust [86] to 18.32 MJ kg^−1^ for crushed pine cones [7]. Similar values were noted in the present study.

El-Sayed and Khairy [87] pelletized powdered corn cob with 40% addition of wheat dust as a binder and found that binder addition increased the HHV of pellets. In the cited study, fixed carbon content was 18% in raw corn cobs and 8.3% in raw wheat dust, respectively, and it increased to 29% for corn cobs and 15.8% for wheat dust after pelleting. As a result, the HHV increased from 16.6 (MJ kg^−1^) to 24.92 (MJ kg^−1^) for corn cobs, and from 14.61 (MJ kg^−1^) to 25.35 (MJ kg^−1^) for wheat dust.

The nitrogen and total carbon content of rye bran and sage straw were determined in the last test, and the results are presented in Table 4.

For rye bran:

Hydrogen—6.0 ± 0.53 (based on the following results: 6.34, 6.27, 5.38);

Sulfur—0.33 ± 0.01 (based on the following results: 0.34, 0.33, 0.32);

Ash—3.78 ± 0.06 (based on the following results: 3.83, 3.71, 3.81).

The average total carbon content was 46.32% in rye bran and 34.92% in sage waste biomass, whereas the average nitrogen content was 2.45% in rye bran and 1.72% in sage. It should be noted that total carbon and nitrogen content was significantly higher in rye bran than in sage waste biomass.

The values noted in rye bran were similar to those reported by Čajová Kantová et al. [88] who analyzed spruce sawdust, spruce bark, and pine cones. In the cited study, carbon content was determined at 46.25–47.26%, hydrogen content—at 6.26–6.36%, and nitrogen content—at 0.24-0.32%. In the work of Zhang et al. [89], the carbon content of spruce-pine-fir sawdust reached 46.2%.

### 3.2. Pelletization Process and Pellet Characteristics

The effect of rye bran addition on the pelletization process of sage waste biomass and the quality (properties) of the obtained pellets is summarized in Table 5. Power/energy consumption from 3.75 kW/107 kWh t^−1^ (0% rye bran content) to 3.19 kW/91 kWh t^−1^.

#### 3.2.1. Energy Consumption

**Figure 3:**
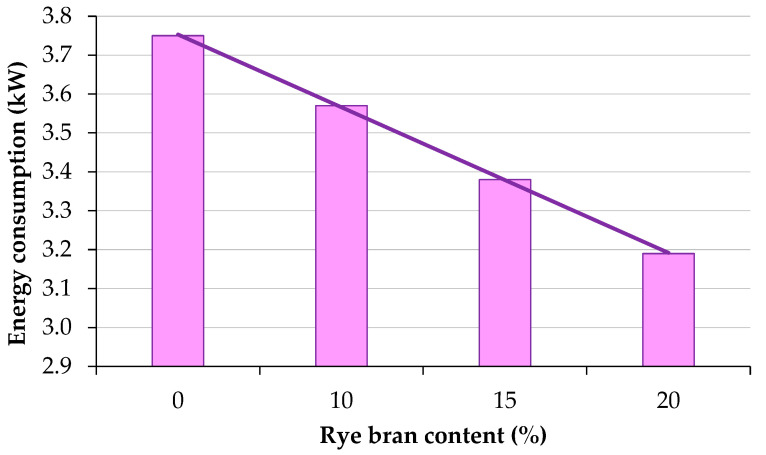
Energy consumption during the pelletization of sage waste biomass with different rye bran content.

The results of energy consumption measurements are presented in Figure 3. Energy consumption was highest during the pelletization of sage waste biomass without the additive. Energy consumption decreased linearly with a rise in rye bran content. The amount of energy required to pelletize sage straw with 20% rye bran addition was equivalent to 85% of energy consumption during the pelletization of pure sage straw.

The influence of rye bran *z_b_* on the energy consumption of the pellet mill *N_g_* during the compaction of the sage straw and rye bran mixture in a flat die pelletizer was determined using Equation (7):

(7) $$N_{g}=-0.187\;z_{b}+3.940$$

where:

*z_b_*—rye bran content of the mixture (%).

The obtained results indicate that rye bran acts as a binder. The binding capacity of rye bran increased with a rise in rye bran content (the additive absorbed water present in the mixture). Rye bran had a lubricating effect on the surface of die openings in the pelletizer, thus reducing the resistance of the material passing through the openings and, consequently, decreasing the mill’s energy consumption (Figure 3). In other studies, binder addition to biomass also reduced energy consumption during the pelletization process. Szyszlak-Bargłowicz et al. [59] reported that 10%, 30%, and 50% addition of copra cake during the production of miscanthus biomass pellets significantly decreased energy consumption from 84.45 Wh kg^−1^ to 39.09 Wh kg^−1^. Garcia et al. [90] pelletized pine sawdust with eleven alternative residual biomasses in a pilot-scale pelletizer and observed that the addition of alternative biomass feedstocks to pine sawdust reduced energy consumption during industrial pelletization. Cui et al. [91] investigated the ability of a microalgae binder to improve the energy consumption and the physical and thermal properties of a novel pellet. They found that the addition of microalgae reduced energy consumption during pelletization by 23.5–40.4%. Tumuluru [92] found that the blending of woody and herbaceous biomass improved pellet quality and decreased energy consumption. The cited author pelletized pine and switchgrass biomass and reported a decrease in energy consumption when larger quantities of pine biomass were added to the mixture. An increase in the content of 2-inch pine residues decreased specific energy consumption to around 90 kWh ton^−1^, whereas an increase in the moisture content of the blend increased specific energy consumption.

Higher rye bran content induced a minor decrease in pellet density. Despite the above, an increase in rye bran content improved PDI values, thus decreasing energy consumption during pelletization and improving pelletization effectiveness.

#### 3.2.2. Physical and Bulk Density of Pellets

**Figure 4:**
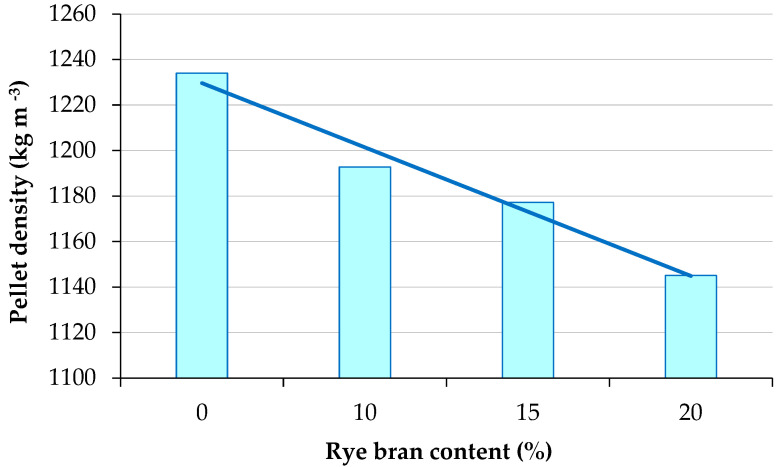
Physical density of sage straw pellets with different rye bran content.

An analysis of pellet density revealed that rye bran addition significantly had a significant effect on the physical density of pellets. The results of measurements are presented in Figure 4 and Table 5. The physical density of pellets decreased significantly with a rise in rye bran content (from 0% to 20%), from 1233.99 kg m^−3^ in pellets containing 0% rye bran to 1145.05 kg m^−3^ in pellets with 20% rye bran addition. The influence of rye bran content *z_b_* on the density *ρ* of the resulting pellets was calculated using Equation (8):

(8) $$\rho =-28.240\;z_{b}+1257.900$$

In other studies, the addition of a binder to compacted biomass also improved the physical properties of pellets. Emadi et al. [93] produced pellets from torrefied non-ground wheat and barley straw with the addition of low-density polyethylene (LDPE) (1, 3, 6 and 10%) as a binder, and found that 6% LDPE addition induced the greatest increase in the density of wheat (1.8%) and barley pellets (1.7%). The tensile strength of wheat and barley pellets increased by 280% and 253%, respectively, when 10% LDPE was added to the mixture. Cui et al. [91] reported that the addition microalgae binder to wood waste biomass increased the bulk density and mechanical strength of pellets by 9–36% and 0.7–1.6%, respectively. Souček and Jasinskas [94] pelletized flax stalks with the addition of potatoes as the binding agent. They observed that potatoes improved the mechanical properties of pellets and reduced carbon monoxide emissions. The addition of potatoes also increased specific weight (599.2 kg m^−3^ in pellets without potatoes and 1092.3 kg m^−3^ in pellets with potatoes) and significantly improved the mechanical strength of the pellets (4.39% in pellets without potatoes and 0.71% in pellets with potatoes).

**Figure 5:**
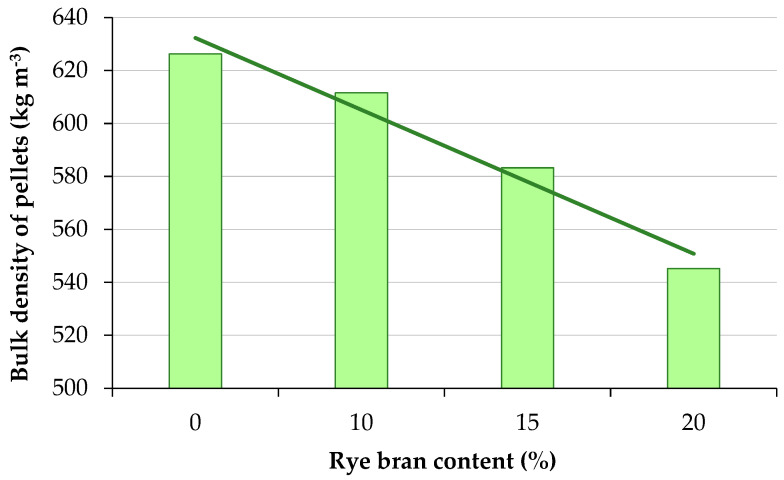
Bulk density of sage straw pellets with different rye bran content.

Rye bran addition caused a similar improvement in the bulk density of pellets (Figure 5). Higher rye bran content induced a minor decrease in pellet density. Despite the above, an increase in rye bran content improved PDI values, thus decreasing energy consumption during pelletization and improving pelletization effectiveness. The influence of rye bran content *z_b_* on the bulk density *ρ_b_* of the resulting pellets was calculated using Equation (9):

(9) $$\rho_{b}=-27.156\;z_{b}+659.430$$

Bulk density was highest at 626.24 kg m^−3^ in pellets composed solely of sage straw, and it decreased to 611.54 kg m^−3^ in pellets containing 10% rye bran and to 547.16 kg m^−3^ in pellets containing 20% rye bran. Bulk density was nearly 13% lower in pellets with 20% rye bran addition than in pellets made from sage straw alone. Bulk density decreased with a rise in rye bran content. The bulk density of pellets composed solely of sage straw was comparable to that of oak sawdust pellets (624.5 kg m^−3^), and the bulk density of pellets containing 15% rye bran was similar to pellets made from willow harvested over a one-year cycle (583.22 kg m^−3^) [57].

#### 3.2.3. Pellet Durability Index

**Figure 6:**
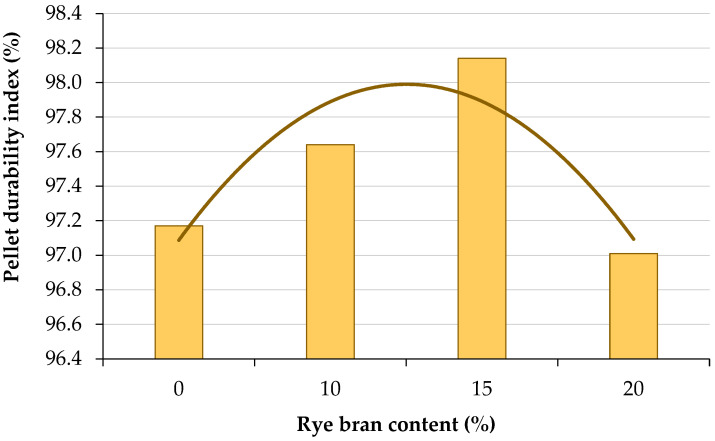
Durability index of sage straw pellets with different rye bran content.

The PDI was highest at 98.14% in pellets with 15% rye bran addition (Figure 6). PDI values were similar in pellets composed solely of sage straw (97.17%) and pellets with 20% rye bran addition (97.01%). The evaluated parameter was markedly higher in pellets containing 10% rye bran (97.64%). An analysis of the Order 2 polynomial trendline revealed the highest PDI values in pellets with 10% and 15% rye bran content. The influence of higher rye bran content on PDI values was most effectively described by the Order 2 polynomial trendline. The coefficient of determination R^2^ was much lower when a linear trendline was used. The rapid decrease in PDI values in pellets containing more than 15% of rye bran could be attributed to a decrease in physical density. Pellets lose their cohesiveness when a certain physical density limit is exceeded.

The influence of rye bran *z_b_* on the PDI of pellets produced from sage waste biomass in a flat die pelletizer was determined using Equation (10):

(10) $$P_{di}=-0.400\;z_{b}^{2}+2.002\;z_{b}+95.485$$

Carrillo-Parra et al. [56] produced pellets from mixtures of oil palm residues and pine sawdust at ratios of 100:0, 80:20, 60:40, 40:60, 20:80, and 0:100, and found that the addition of pine sawdust improved selected mechanical properties of agro-pellets. According to Tumuluru [92], a higher content of pine in pelleted switchgrass blends increased PDI values. These results were confirmed by Brand and Jacinto [95] who reported that the pine wood with apple pruning mix used for pellet production reduced ash content, increased bulk density and energy density, and improved the quality of pellets. Espuelas et al. [96] analyzed the suitability of spent coffee grounds (SCG) for briquette production with xanthan and guar gums as binders and found that dry density was highest (0.819 g cm^−3^) in briquettes manufactured with 10% addition of xanthan (15% moisture content, compaction pressure—12 MPa). A combination involving 5% addition of xanthan (30% moisture content, compaction pressure—12 MPa) was most durable, with a mass loss of only 3.9%. However, binders exerted a different effect on the properties of biofuel pellets manufactured from yak manure by Liu et al. [97]. The tested binders did not positively affect the density or the diametral compressive strength of pellets.

In the present study, pellets with 15% rye bran content were characterized by a high PDI (98.14%) which was comparable to the values noted by Kraszkiewicz et al. [98] in pellets made from straw, grain mix (98.33%) or meadow hay (97.67%). In this study, the PDI values of pellets without rye bran or with 20% rye bran addition reached 97.17% and 97.01%, respectively, and were comparable with rye and wheat straw pellets.

## 4. Conclusions

In recent years, various waste biomass materials, including agricultural waste, garden waste, food industry waste, and herbal waste, have been used for heat generation. Sage straw, as a by-product of sage production, is one type of herbal waste biomass that can be used for this purpose.

In this study, sage straw with the addition of rye bran (10%, 15%, and 20%) was pelletized in the SS-4 test stand using a P-300 pelletizer with flat die roller compactors. The study demonstrated that rye bran addition had a significant effect on the pelletizer’s energy consumption, the quality of the obtained pellets (physical density, bulk density, PDI), and the HHV and LHV of pellets.

An increase in rye bran content decreased the pelletizer’s energy consumption and increased PDI values, but it exerted a negative impact on pellet quality (decrease in physical density and bulk density) and decreased the HHV and LHV of pellets. Pellets containing sage straw only were characterized by the highest LHV (17.023), but had a low PDI (97.17%); therefore, the addition of rye bran can improve the quality of the resulting product. Even small quantities of rye bran increase the mechanical strength of pellets and reduce the pelletizer’s energy consumption.

## Figures and Tables

**Table 1:** Higher heating value and lower heating value of sage straw.

No.	Moisture Content (%)	Higher Heating Value (MJ/kg)		Lower Heating Value (MJ/kg)	
		Wet Basis	Dry Basis	Wet Basis	Dry Basis
1	10.1	19.347	21.502	18.122	20.410
2		19.415	21.575	18.188	20.483
3		19.553	21.728	18.326	20.437
4		19.270	21.414	18.043	20.322
Average	10.1	19.396	21.554	18.169	20.413

**Table 2:** Higher heating value and lower heating value of rye bran.

No.	Moisture Content (%)	Higher Heating Value (MJ/kg)		Lower Heating Value (MJ/kg)	
		Wet Basis	Dry Basis	Wet Basis	Dry Basis
1	13.2	16.850	19.409	15.196	17.874
2		16.714	19.279	15.061	17.718
3		16.738	19.358	15.084	17.745
4		16.808	19.305	15.154	17.824
Average	13.2	16.778	19.320	15.124	17.790

**Table 3:** Higher heating value and lower heating value of sage straw pellets with different rye bran content.

Content of Rye Bran (%)	Higher Heating Value (MJ kg^−1^)	Lower Heating Value (MJ kg^−1^)
	15% Moisture Content	15% Moisture Content
0	18.316	17.023
5	18.221	16.909
10	18.126	16.796
15	18.031	16.682
20	17.936	16.569

**Table 4:** Nitrogen and total carbon content of rye bran and sage straw.

No.	Rye Bran		Sage Straw	
	Nitrogen (%)	Total Carbon (%)	Nitrogen (%)	Total Carbon (%)
1	2.41	46.33	1.79	35.23
2	2.45	46.33	1.75	34.34
3	2.48	46.30	1.83	35.20
Average	2.45	46.32	1.79	34.92
SD (%)	0.035	0.017	0.040	0.510

**Table 5:** The effect of rye bran addition on the pelletization process of sage waste biomass and the quality of the obtained pellets.

Bran Content (%)	Pelletizer’s Power/Energy Consumption (kW/kWh t^−1^)	Physical Density (kg m^−3^)	Bulk Density (kg m^−3^)	PDI (%)
0	3.75/107	1233.99	626.24	97.17
10	3.57/102	1192.80	611.54	97.64
15	3.38/96	1177.18	583.22	98.14
20	3.19/91	1145.05	545.16	97.01

## Data Availability

Not applicable.

## References

[1] Perspektywy Rozwoju Energii Odnawialnej W Polsce. https://www.irena.org/-/media/Files/IRENA/Agency/Publication/2015/IRENA_REmap_Poland_paper_2015_PL.PDF?la=en&hash=37E52205C649C5FF87FB1DAC3127ABF5B5FA35E5.

[2] (2022). Renewables 2022. Global Status Report. https://www.ren21.net/wp-content/uploads/2019/05/GSR2022_Full_Report.pdf.

[3] eia U.S. Energy Information Administration. https://www.eia.gov/energyexplained/biomass.

[4] Azócar L., Hermosilla N., Gay A., Rocha S., Díaz J., Jara P. (2019). Brown pellet production using wheat straw from southern cities in Chile. Fuel.

[5] Obidziński S., Hejft R., Dołżyńska M. (2017). Study of cereal waste granulation process. Przem. Chem..

[6] Serrano C., Monedero E., Lapuerta M., Portero H. (2011). Effect of moisture content, particle size and pine addition on quality parameters of barley straw pellets. Fuel Process. Technol..

[7] Gendek A. (2015). Combustion heat and calorific value of the mix of sawdust and cones of common pine (*Pinus sylvestris* L.). Ann. Wars. Univ. Life Sci. Agric. (Agric. For. Eng.).

[8] Stasiak M., Molenda M., Bańda M., Wiącek J., Parafiniuk P., Gondek E. (2017). Mechanical and combustion properties of sawdust—Straw pellets blended in different proportions. Fuel Process. Technol..

[9] Thiffault E., Barrette J., Blanchet P., Nguyen Q.N., Adjalle K. (2019). Optimizing quality of wood pellets made of hardwood processing residues. Forests.

[10] Tenorio C., Moya R., Tomazello-Filho M., Valaert J. (2015). Quality of pellets made from agricultural and forestry crops in Costa Rican tropical climates. BioResources.

[11] Pradhan P., Arora A., Mahajani S.M. (2018). Pilot scale evaluation of fuel pellets production from garden waste biomass. Energy Sustain. Dev..

[12] Obidziński S., Dołżyńska M., Kowczyk-Sadowy M., Jadwisieńczak K., Sobczak P. (2019). Densification and fuel properties of onion husks. Energies.

[13] Dołżyńska M., Obidziński S., Kowczyk-Sadowy M., Krasowska M. (2019). Densification and combustion of cherry stones. Energies.

[14] Kobus Z., Panasiewicz M., Zawiślak K., Sobczak P., Mazur J., Guz T., Nadulski R. (2014). Analysis of possibilities of obtaining essential oils from herbaceous plants waste. Inżynieria Rol..

[15] Kumar M., Kumar V., Roy D., Kushwaha R., Vaiswani S. (2014). Application of herbal feed additives in animal nutrition—A review. Int. J. Livest. Res..

[16] Raal A., Orav A., Püssa T., Valner C., Malmiste B., Arak E. (2012). Content of essential oil, terpenoids and polyphenols in commercial chamomile (*Chamomilla recutita* L. Rauschert) teas from different countries. Food Chem..

[17] Suganya T., Senthilkumar S., Deepa K., Muralidharan J., Gomathi G., Gobiraju S. (2016). Herbal feed additives in poultry. Int. J. Sci. Environ. Technol..

[18] Nowak D., Syta M. (2009). Identification of the impact of grinding degree, pretreatment and drying method on content of betalaine dyes in dried beet material. Inżynieria Rol..

[19] Czubaszek R. (2019). The assessment of the suitability af lemon balm and alder buckthorn wastes for the biogas production. J. Ecol. Eng..

[20] Lee Y., Kim S., Kim J., Shin G.-A., Lee C.-G., Seungho Jung S., Lee J. (2020). Catalytic pyrolysis as a technology to dispose of herbal medicine waste. Catalysts.

[21] Haq T., Begum T., Ali T.A., Iqbal S., Khan F.A. (2016). Recycling of biomass waste from herbal pharmaceutical industry by windrow composting. Int. J. Sci. Res. Publ..

[22] Kowalski R., Wawrzykowski J. (2009). Essential oils analysis in dried materials and granulates obtained from *Thymus vulgaris* L., *Salvia officinalis* L., *Mentha piperita* L. and *Chamomilla recutita* L. Flavour Fragr. J..

[23] Das V., Satyanarayan S., Satyanarayan S. (2017). Recycling of recalcitrant solid waste from herbal pharmaceutical industry through vermicomposting. Int. J. Environ. Agric. Biotech..

[24] Sienkiewicz A., Piotrowska-Niczyporuk A., Bajguz A. (2020). Fatty acid methyl esters from the herbal industry wastes as a potential feedstock for biodiesel production. Energies.

[25] Kowalska H. (2018). Sustainable technologies—Utilization of plant by-products. Przemysł Spożywczy.

[26] Lewicki A., Pilarski K., Janczak D., Czekała W., Rodríguez Carmona P.C., Cieślik M., Witaszek K., Zbytek Z. (2013). The biogas production from herbs and waste from herbal industry. J. Res. Appl. Agric. Eng..

[27] Haghighi M., Afsharikia A., Mozafariyan M., Pessarakli M., Bolandnazar A. (2014). Usage of herbal (thyme and chicory) waste as an organic substrate in cucumber production. Commun. Soil Sci. Plant Anal..

[28] Obidziński S., Joka M., Fijoł O. (2017). Two-stage agglomeration of fine-grained herbal nettle waste. Int. Agrophysics.

[29] Tulska E. (2018). Możliwości Zagospodarowania Surowców Odpadowych do Produkcji Opakowań.

[30] Kisworo A.N., Agus A., Kustantinah K., Suwignyo B. (2016). Physicochemical characteristics identification and secondary metabolite analysis of solid herbal waste as source of feed rich fiber and supplement for ruminants. Anim. Prod..

[31] Zhuang X., Chen Z., Sun X., Li F., Luo J., Chen T., Xi Q., Zhang Y., Sun J. (2021). Fermentation quality of herbal tea residue and its application in fattening cattle under heat stress. BMC Vet. Res..

[32] Kowczyk-Sadowy M., Piekut J., Obidziński S. (2018). The effect of potato pulp addition on the compaction of couch grass mixture. Przem. Chem..

[33] Teixeira E.M.B., Carvalho M.R.B., Neves V.A., Silva M.A., Arantes-Pereira L. (2014). Chemical characteristics and fractionation of proteins from *Moringa oleifera* Lam. leaves. Food Chem..

[34] Kongmun P., Wanapat M., Pakdee P., Navanukraw C., Yu Z. (2011). Manipulation of rumen fermentation and ecology of swamp buffalo by coconut oil and garlic powder supplementation. Livest. Sci..

[35] Yang W.Z., Benchaar C., Ametaj B.N., Chaves A.V., He M.L., McAllister T.A. (2007). Effects of garlic and juniper berry essential oils on ruminal fermentation, site and extent of digestion in lactating cows. J. Dairy Sci..

[36] Maenner K., Vahjen W., Simon O. (2011). Studies on the effects of essential-oil-based feed additives on performance, ileal nutrient digestibility and selected bacterial groups in the gastrointestinal tract of piglets. J. Anim. Sci..

[37] El Tazi S.M.A., Mukhtar M.A., Mohamed K.A., Tabidi M.H. (2014). Effect of using black pepper as natural feed additive on performance and carcass quality of broiler chicks. Glob. Adv. Res. J. Agric. Sci..

[38] Singh D., Suthar S. (2012). Vermicomposting of herbal pharmaceutical industry waste: Earthworm growth, plant-available nutrient and microbial quality of end materials. Bioresour. Technol..

[39] Zhang J.C., Zeng G.M., Chen Y.N., Yu M., Yu Z., Li H., Yu Y., Huang H. (2011). Effects of physico-chemical parameters on the bacterial and fungal communities during agricultural waste composting. Bioresour. Technol..

[40] Zhang X.X., Wang B.Y., Liu Z.W. (2019). Impacts of plant secondary metabolites from conifer litter on the decomposition of *Populus purdomii* litter. J. For. Res..

[41] Zhou Y., Selvam A., Wong J.W.C. (2016). Effect of Chinese medicinal herbal residues on microbial community succession and anti-pathogenic properties during co-composting with food waste. Bioresour. Technol..

[42] Zhou Y., Selvam A., Wong J.W.C. (2018). Chinese medicinal herbal residues as a bulking agent for food waste composting. Bioresour. Technol..

[43] Greff B., Szigeti J., Varga A., Lakatos E., Sáhó A., Varga L. (2021). Effect of bacterial inoculation on co composting of lavender (*Lavandula angustifolia* Mill.) waste and cattle manure. 3 Biotech.

[44] Najda A. (2017). Zmienność Ontogenetyczna Mięty (Mentha Species) Czynnikiem Warunkującym Zawartość Składników Bioaktywnych w Surowcu.

[45] Rokicki T., Golonko M. (2017). Foreign trade of herbs and spices in the world. Zesz. Nauk. SGGW W Warszawie—Probl. Rol. Swiat..

[46] Newerli-Guz J. (2016). The cultivation of herbal plants in Poland. Rocz. Nauk. Stowarzyszenia Ekon. Rol. I Agrobiz..

[47] Wrzesińska-Jędrusiak E., Klimek K., Najda A., Łaska-Zieja B., Olesienkiewicz A. (2020). Study on the potential of biogas production from herbal residues. Przem. Chem..

[48] Ubwa S.T., Asemave K., Oshido B., Idoko A. (2013). Preparation of biogas from plants and animal waste. Int. J. Sci. Technol..

[49] Obidziński S. (2012). Pelletization process of postproduction plant waste. Int. Agrophysics.

[50] Obidziński S. (2013). Profile of water activity and geometric parameters of lemon balm wastes in the aspect of their utilisation as an addition to fodders. Acta Agrophysica.

[51] Gill N., Dogra R., Dogra B. (2018). Influence of moisture content, particle size, and binder ratio on quality and economics of rice straw briquettes. BioEnergy Res..

[52] Atabani A.E., Mahmoud E., Aslam M., Naqvi S.R., Juchelková D., Bhatia S.K., Badruddin I.A., Khan T.M.Y., Hoang A.T., Palacky P. (2022). Emerging potential of spent coffee ground valorization for fuel pellet production in a biorefinery. Environ. Dev. Sustain..

[53] Blancarte-Contreras E., Corral-Rivas S., Domínguez-Gómez T.G., Lujan-Soto J.E., Goche-Télles J.R., Montiel-Antuna E. (2022). Improving the physical, mechanical and energetic characteristics of pine sawdust by the addition of up to 40% *Agave durangensis* gentry pellets. Energies.

[54] Chojnacki J., Zdanowicz A., Ondruška J., Šooš Ľ., Smuga-Kogut M. (2021). The Influence of apple, carrot and red beet pomace content on the properties of pellet from barley straw. Energies.

[55] Kulokas M., Praspaliauskas M., Pedišius N. (2021). Investigation of buckwheat hulls as additives in the production of solid biomass fuel from straw. Energies.

[56] Carrillo-Parra A., Contreras-Trejo J.C., Pompa-García M., Pulgarín-Gámiz M.A., Rutiaga-Quiñones J.G., Pámanes-Carrasco G., Ngangyo-Heya M. (2020). Agro-pellets from oil palm residues/pine sawdust mixtures: Relationships of their physical, mechanical and energetic properties, with the raw material chemical structure. Appl. Sci..

[57] Stolarski M., Szczukowski S., Tworkowski J., Kwiatkowski J., Grzelczyk M. (2005). Characteristic of chips and pellets from the *Coppice willow* and *Virginia mallow* biomass as a fuel. Probl. Inżynierii Rol..

[58] Gonzalez W.A., Lopez D., Perez J.F. (2020). Biofuel quality analysis of fallen leaf pellets: Effect of moisture and glycerol contents as binders. Renew. Energy.

[59] Szyszlak-Bargłowicz J., Słowik T., Zając G., Blicharz-Kania A., Zdybel B., Andrejko D., Obidziński S. (2021). Energy parameters of miscanthus biomass pellets supplemented with copra meal in terms of energy consumption during the pressure agglomeration process. Energies.

[60] Harasym J. (2011). Biorafinacja ziarna zbożowego. Przegląd Zbożowo Młynarski.

[61] Arendt E.K., Zannini E. (2013). Cereal grains for the food and beverage industries. Woodhead Publ. Ser. Sci. Technol. Nutr..

[62] Kamal-Eldin A., Nygaard Laerke H., Bach Knudsen K.-E., Lampi A.-M., Piironen V., Adlercreutz H., Åman P. (2009). Physical, microscopic and chemical characterisation of industrial rye and wheat brans from the Nordic countries. Food Nutr. Res..

[63] Delcour J.A., Poutanen K. (2013). Fibre-Rich and Wholegrain Foods. A Volume in Woodhead Publishing Series in Food Science, Technology and Nutrition.

[64] Śmiechowska M., Jurasz M. (2014). Zawartość włókna surowego w wybranych produktach zbożowych. Probl. Hig. I Epidemiol..

[65] Obidziński S., Dołżyńska M., Stasiełuk W. (2019). Production of fuel pellets from a mixture of sawdust and rye bran. IOP Conference Series: Earth and Environmental Science.

[66] Szyszlak-Bargłowicz J., Piekarski W., Słowik T., Zając G., Krzaczek P., Sobczak P. (2011). Właściwości mechaniczne peletów z biomasy ślazowca pensylwańskiego. Autobusy: Tech. Eksploat. Syst. Transp..

[67] Zając G., Szyszlak-Bargłowicz J. (2011). Wpływ dodatku otrąb żytnich na własności energetyczne peletów z biomasy ślazowca pensylwańskiego. Autobusy: Tech. Eksploat. Syst. Transp..

[68] (2015). Solid Biofuels—Determination of Moisture Content. Drier Method. Part 1: Total Moisture—Reference Method.

[69] (2009). Fodder—Determination of the Grinding.

[70] (2020). Solid Fuels. Determination of Heat of Combustion by Combustion in a Calorimeter Bomb and Calculation of Calorific Value.

[71] Kordylewski W. (2008). Spalanie i Paliwa.

[72] (2016). Solid Biofuels—Determination of the Bulk Density.

[73] (2016). Solid Biofuels—Determination of Mechanical Durability of Pellets and Briquettes. Part 1: Pellets.

[74] Harun N.Y., Afzal M.T. (2016). Effect of particle size on mechanical properties of pellets made from biomass blends. Procedia Eng..

[75] Jacinto R.C., Brand M.A., da Cunha A.B., Souza D.L., da Silva M.V. (2017). Use of waste from the production chain of pinion for the production of pellets for energy generation. Floresta.

[76] Pradhan P., Mahajani S.M., Arora A. (2021). Pilot scale production of fuel pellets from waste biomass leaves: Effect of milling size on pelletization process and pellet quality. Fuel.

[77] Stelte W., Holm J.K., Sanadi A.R., Barsberg S., Ahrenfeldt J., Henriksen U.B. (2011). Fuel pellets from biomass: The importance of the pelletizing pressure and its dependency on the processing conditions. Fuel.

[78] Mani S., Tabil L.G., Sokhansanj S. (2006). Effects of compressive force, particle size and moisture content on mechanical properties of biomass pellets from grasses. Biomass Bioenergy.

[79] Carone M.T., Pantaleo A., Pellerano A. (2011). Influence of process parameters and biomass characteristics on the durability of pellets from the pruning residues of *Olea europaea* L. Biomass Bioenergy.

[80] Jasinskas A., Streikus D., Vonžodas T. (2020). Fibrous hemp (Felina 32, USO 31, Finola) and fibrous nettle processing and usage of pressed biofuel for energy purposes. Renew. Energy.

[81] Miranda M.T., Sepúlveda F.J., Arranz J.I., Montero I., Rojas C.V. (2018). Analysis of pelletizing from corn cob waste. J. Environ. Manag..

[82] Gageanu I., Voicu G., Vladut V., Voicea I. (2017). Experimental research on influence of recipes used on quality of biomass pellets. Eng. Rural. Dev..

[83] Nakomcic-Smaragdakis B., Cepic Z., Dragutinovic N. (2016). Analysis of solid biomass energy potential in Autonomous Province of Vojvodina. Renew. Sustain. Energy Rev..

[84] Obidziński S., Puchlik M., Dołżyńska M. (2020). Pelletization of post-harvest tobacco waste and investigation of flue gas emissions from pellet combustion. Energies.

[85] Dołżyńska M., Obidziński S., Piekut J., Yildiz G. (2020). The utilization of plum stones for pellet production and investigation of post-combustion flue gas emissions. Energies.

[86] Gendek A., Aniszewska M., Malat’ák J., Velebil J. (2018). Evaluation of selected physical and mechanical properties of briquettes produced from cones of three coniferous tree species. Biomass Bioenergy.

[87] El-Sayed S.A., Khairy M. (2017). Preparation and characterization of fuel pellets from corn cob and wheat dust with binder. Iran. J. Energy Environ..

[88] Čajová Kantová N., Holubčík M., Čaja A., Trnka J., Jandačka J. (2022). Analyses of pellets produced from spruce sawdust, spruce bark, and pine cones in different proportions. Energies.

[89] Zhang B., Yang B., Wu S., Guo W., Zhang J., Wu Z., Wang Z., Lim J.C. (2021). Effect of torrefaction pretreatment on the fast pyrolysis behavior of biomass: Product distribution and kinetic analysis on spruce-pin-fir sawdust. J. Anal. Appl. Pyrolysis.

[90] Garcia R., Gil M.V., Rubiera F., Pevida C. (2019). Pelletization of wood and alternative residual biomass blends for producing industrial quality pellets. Fuel.

[91] Cui X., Yang J., Shi X., Lei W., Huang T., Bai C. (2019). Experimental investigation on the energy consumption, physical, and thermal properties of a novel pellet fuel made from wood residues with microalgae as a binder. Energies.

[92] Tumuluru J.S. (2019). Pelleting of pine and switchgrass blends: Effect of process variables and blend ratio on the pellet quality and energy consumption. Energies.

[93] Emadi B., Iroba K.L., Tabil L.G. (2017). Effect of polymer plastic binder on mechanical, storage and combustion characteristics of torrefied and pelletized herbaceous biomass. Appl. Energy.

[94] Souček J., Jasinskas A. (2020). Assessment of the use of potatoes as a binder in flax heating pellets. Sustainability.

[95] Brand M.A., Jacinto R.C. (2020). Apple pruning residues: Potential for burning in boiler systems and pellet production. Renew. Energy.

[96] Espuelas S., Marcelino S., Echeverría A.M., Castillo J.M., Seco A. (2020). Low energy spent coffee grounds briquetting with organic binders for biomass fuel manufacturing. Fuel.

[97] Liu J., Jiang X., Yuan Y., Chen H., Zhang W., Cai H., Gao F. (2022). Densification of yak manure biofuel pellets and evaluation of parameters: Effects on properties. Energies.

[98] Kraszkiewicz A., Kachel-Jakubowska M., Szpryngiel M., Niedziółka I. (2013). The analysis of the selected quality properties of pellets made of plant raw materials. Inżynieria Rol..

